# Equity of expenditure changes associated with a sweetened-beverage tax in Tonga: repeated cross-sectional household surveys

**DOI:** 10.1186/s12889-020-10139-z

**Published:** 2021-01-18

**Authors:** Andrea Teng, Bertrand Buffière, Murat Genç, Telekaki Latavao, Viliami Puloka, Louise Signal, Nick Wilson

**Affiliations:** 1 Department of Public Health, University of Otago, PO Box 7343, Wellington, New Zealand; 2 Pacific Community, Nouméa, New Caledonia; 3 Department of Economics, University of Otago, Dunedin, New Zealand; 4 Department of Statistics, Nuku’alofa, Tonga

**Keywords:** Expenditure, Sweetened-beverages, Heterogeneity, Inequalities, Excise tax, Tonga

## Abstract

**Background:**

The aim of this study was to examine changes in beverage expenditure patterns before and after a T$0.50/L sweetened-beverage (SB) excise was introduced in Tonga in 2013, by household income, household age composition and island of residence.

**Methods:**

Two cross-sectional surveys involved households being randomly sampled (the Household Income and Expenditure Surveys in 2009 (*n* = 1982) and 2015/16 (*n* = 1800)). Changes in soft drink (taxed), bottled water, and milk (both untaxed) expenditure were examined namely: (i) prevalence of households purchasing the beverage; (ii) average expenditure per person (inflation-adjusted); (iii) expenditure as a proportion of household food budget; and (iv) expenditure per person as a proportion of equivalised income.

**Results:**

The pattern found was of decreases in all soft drink expenditure outcomes and these appeared to be greater in low-income than high-income households for purchasing prevalence (− 30% and − 25% respectively, t-test *p* = 0.98), per-capita expenditure (− 37% and − 34%, *p* = 0.20) and food budget share (− 27% and − 7%, *p* = 0.65), but not income share (− 6% and − 32%, *p* = 0.71). The large expenditure increases in bottled water appeared to be greater in low-income than high-income households for purchasing prevalence (355 and 172%, *p* = 0.32) and food budget share (665 and 468%, *p* = 0.09), but greater in high-income households for per-capita expenditure (121 and 373%, *p* < 0.01) and income share (83 and 397%, *p* = 0.50).

**Conclusions:**

The sweetened-beverage tax was associated with reduced soft drink purchasing and increased bottled water expenditure. Low-income households appeared to have slightly greater declines in soft drink expenditure.

**Supplementary Information:**

The online version contains supplementary material available at 10.1186/s12889-020-10139-z.

## Background

Sugar-sweetened beverage (SSB) taxes have been shown to reduce purchasing and dietary intake of taxed beverages across jurisdictions [1]. However, it is unclear to what extent reductions vary by socioeconomic position (SEP) and other factors such as age, obesity, rurality and level of consumption. Heterogeneity is important because a key public health goal in many settings is to improve health equity and there is interest in which interventions work best for people with the highest health and financial needs. Modelling studies suggest SSB taxes can have both progressive health effects (pro-health equity), and also be financially regressive [2, 3], for example low-income households tend to pay more as a proportion of their income or expenditure. However, SSB taxes may typically reduce expenditure on SSBs more for low SEP than high SEP households, owing to greater price sensitivities of low-income groups. The overall fiscal burden and health benefit of SSB taxes on low-income households depends on how much consumption decreases and on substitution patterns [4], particularly to other beverages. The tax burden is typically very small and has been found to be similar in low- and high-income households [2, 5]. Earmarked revenue from SSB taxes into other health interventions may also subsequently impact on equity, as per various jurisdictions [6].

In jurisdictions that have introduced SSB taxes and evaluated them by SEP, the pattern of soft drink consumption has been mixed. Evaluations of a 1 peso/L SSB tax in Mexico have reported significantly greater SSB tax effects (% change) on per capita purchased volumes in groups with low SEP [7, 8]. Results were similar from evaluations of sales taxes in the United States where children from low-income families had larger declines in dietary intake as a result of differential state soft drink sales taxes [9]. In Chile, conversely, after a relatively small SSB tax increase from 13 to 18% in beverages with ≥ 6.25 g sugar/100 ml, two evaluations reported that the largest declines in taxed beverage purchase volumes were in high SEP groups [10, 11]. This could have been due to non-tax factors such as media coverage of the tax or other policies such as front of pack labelling [12] having more impact on better educated consumers [13]. In Catalonia, Spain, a €0.12/L SSB tax on beverages with > 8 g sugar/100 ml, was associated with greater declines in high-income regions [14], and high-income households in a second study [15], who had greater reductions in soft drink expenditure shares and grams of sugar from soft drinks [15].

Substitution impacts of SSB taxes to untaxed beverages may also vary by SEP but few real world studies have investigated this. In Mexico the post-tax increase (% change) in bottled water expenditures was higher in low- and middle-income households [7], and in a second study the highest increase (% change) in untaxed beverages was in middle-socioeconomic households [8]. It remains to be seen if the same pattern is evident in other jurisdictions. This has important potential consequences for health equity.

Variation by context suggests that equity effects may be difficult to predict and detailed country-specific analyses are recommended [4]. Results between jurisdictions may be affected by the size of the tax, extent of pass-through to beverage prices, other non-communicable disease (NCD) policy changes, pre-existing trends in beverage consumption, extent of public health messaging [13], and changes in local production and throughout the food system. Existing studies generally assessed relatively small SSB taxes (< 12%), and larger beverage tax changes such as those in Tonga may be associated with a stronger SEP pattern.

Pacific Island and Small Island Developing State SSB taxes have been rarely studied. Tonga was selected for this study because there was a relatively large 27% point increase in the size of the Tonga sweetened-beverage (SB) tax as a percentage of the import price. In August 2013 [16] a 15% import tariff on SBs was replaced with an excise of T$0.50/L (US$0.28/L, 42% of import value) and subsequently doubled to T$1.00/L in July 2016 (63% of import value). The excise applied to full sugar and artificially sweetened soft drinks, energy drinks, and other SBs coded by trade harmonised system code 22.02. The tax did not apply to water (sparkling or flat), juice (sweetened or unsweetened), powdered juice drinks, tea, coffee or hot chocolate. There was little media attention on the SB excise and awareness of the SB tax was low in 2017 [17]. No other major NCD policies were introduced in 2013, however in July 2016 (and 2017) a number of health food taxes were also introduced. The impact of the excise has been associated with reduced import volumes of taxed beverages [18] and changes in beverage purchasing behaviour (38% respondents in 2017 reported reducing their SB consumption in response to SB tax) [17]. However, it remains unclear which age, income groups and islands experienced the greatest financial costs and declines in consumption from the SB excise.

Tonga is an upper-middle-income country (World Bank) with a high dependence on imported foods (52%) [19] and vulnerability to food insecurity [20]. In 2004 two-thirds of adults in Tonga were obese, which is one of the highest rates of obesity in the world; and 17% of adults had diabetes [21] but this has likely increased subsequently. SBs are commonly consumed by children and adolescents [22] particularly in the main island of Tongatapu (approximately 70% of the population) where consumption has been recorded as fourfold greater than Ha’apai [23], an outer island group. The Tonga census has identified that most households rely on rain water tanks as their main source of drinking water with the majority relying on their own cement tank but a quarter using water from a neighbour [24].

Given this background, the aim of this study was to examine changes in taxed and untaxed beverage expenditure patterns from 2009 to 2015/16 by household income, household age composition and island region; before and after the 2013 Tonga SB excise introduction.

## Methods

### Data

Two cross-sectional surveys were used ie, the 2009 and 2015/16 Tonga Household Income and Expenditure Surveys (HIES). Each survey adopted a two-stage sampling strategy with stratification by island, sampling proportional to census block size and random selection of 12 households from each census block [25, 26]. The 2009 survey was collected at four time points spanning the calendar year, and the 2015/16 survey was collected in 16 rounds over 12 months (4 rounds per quarter) from October 2015 to October 2016 (Table 1). Households surveyed between July and September 2016 (26%) would have also experienced the doubling of the SB tax in to T$1.00/L from beginning of July 2016 [27]. Response rates were high at 96.1% in 2009 and 98.8% in 2015/16. A very small proportion of these households did not report any food expenditures and were excluded from the analysis (0.05 and 0.17% for each survey respectively). Thus, food and beverage expenditure data were recorded by 1982 households in 2009 and 1800 households in 2015/16.

**Table 1:** Changes in characteristics of the Tonga Household Income and Expenditure Survey samples, 2009 to 2015/16

Characteristic		2009		2015/16		Difference
		n	% or mean (95% CI)	n	% or mean (95% CI)	[percentage points (95% CI), absolute difference]
Total households	Sum	1982	100	1800	100	
Household age composition	Adults only	519	26.2 (24.1 to 28.3)	506	28.1 (25.6 to 30.6)	1.9 (−1.3 to 5.2)
	Adults & children	1463	73.8 (71.7 to 75.9)	1294	71.9 (69.4 to 74.4)	
	*Mean age (years)*		*29.4 (28.7 to 30.1)*		*31.6 (30.5 to 32.7)*	*2.2 (0.9 to 3.5)*
Mean equivalised household income by income tertile	*Lowest tertile (T$)*	665	*3739 (3595 to 3883)*	523	*4168 (4041 to 4295)*	*428 (237 to 620)*
	*Middle tertile (T$)*	650	*8358 (8231 to 8485)*	637	*8808 (8646 to 8970)*	*450 (244 to 656)*
	*Highest tertile (T$)*	668	*21,704 (20,286 to 23,122)*	639	*21,906 (19,734 to 24,078)*	*202 (− 2392 to 2796)*
Main drinking water source	Bottled water	39	2.0 (1.2 to 2.7)	144	8.0 (5.7 to 10.3)	6.0 (3.6 to 8.5)
	Tank/other	1943	98.0 (97.3 to 98.8)	1656	92.0 (89.7 to 94.3)	
Island	Tongatapu (main island)	1367	69.0 (68.7 to 69.3)	1292	71.8 (70.4 to 73.1)	2.8 (1.4 to 4.1)
	Outer island/s	615	31.0 (30.7 to 31.3)	508	28.2 (26.9 to 29.6)	
Household size	*Mean persons per household*		*5.2 (5.0 to 5.4)*		*5.7 (5.5 to 5.9)*	*0.5 (0.2 to 0.7)*
Annual quarter	Oct-Dec	474	23.9 (17.3 to 30.5)	595	33.1 (24.2 to 41.9)	9.2 (−1.9 to 20.2)
	Jan-Mar	491	24.7 (18.0 to 31.5)	315	17.5 (10.5 to 24.5)	−7.3 (− 17.0 to 2.5)
	Apr-Jun	529	26.7 (19.9 to 33.5)	427	23.7 (16.2 to 31.2)	−3.0 (−13.2 to 7.1)
	Jul-Sep^a^	488	24.6 (17.9 to 31.4)	460	25.5 (17.5 to 33.6)	0.9 (−9.6 to 11.4)
	Missing			4	0.2 (0.0 to 0.4)	
Household adult obesity	All adults obese	–	–	579	32.2 (28.9 to 35.5)	
	Mixed	–	–	1006	55.9 (52.9 to 58.9)	
	No adults obese	–	–	207	11.5 (9.5 to 13.6)	
	Missing	–	–	8	0.4 (0.1 to 0.8)	
	*Mean BMI (kg/m*^*2*^*)*		*–*		*32.3 (31.9 to 32.6)*	

^a^A further tax increase was introduced at the beginning of July 2016 in the final quarter of the 2015/16 HIES study which started in October 2015. Children were less than 15 years old, and adults were 15 years and older, as categorised in the Tonga HIES survey. All values are survey weighted to be representative of the national Tongan population. Income tertiles were calculated on unweighted data and therefore do not include exactly one-third of households. Source: Tonga Department of Statistics, Household Income and Expenditure Surveys

Each household was asked to keep a diary for 2 weeks recording household expenditure, gifts received and home-produced items. The categorisation of beverages in 2015/16 was more detailed. The categories aligned to the broader 2009 beverage categories for soft drinks, milk and bottled water (see Additional File Table B for definitions and alignment). Households were also asked about the number of household members, ages, income, island, time period (annual quarter) of expenditure, main drinking water source, highest qualification, and (in 2015/16) the height and weight of each household member were collected. Children (less than 15 years old) and adults (15 years and older) were categorised in the same way as the Tonga HIES.

### Outcomes

The dependent variable (outcome) was the level of expenditure on soft drinks (all brands and including artificially-sweetened beverages), milk (dairy products) and bottled water. Of these beverage categories, the 2013 and 2016 SB taxes applied fully to soft drinks. Expenditure was the value of beverages acquired, used or paid for by a household through direct monetary purchases, home production, barter and as income in-kind for consumption by household members. Four measures of expenditure were used: (i) proportion of households acquiring one or more beverages in the 2 week period (purchasing prevalence); (ii) beverage expenditure adjusted for inflation (2015/16 T$ per household member per year); (iii) beverage as a proportion of all food and non-alcoholic beverage expenditures (food budget share, a measure of household food preference [28]); and (iv) beverage expenditure per person as a proportion of equivalised household income (income share). Multiple measures were selected to improve comparability with other studies [4]. All measures were based on the total survey sample, not just beverage consumers.

### Analysis

Average household expenditure outcomes in the 2009 and 2015/16 surveys were compared to assess any changes after the SB tax for taxed and untaxed beverages. The absolute differences in mean expenditure outcomes between the two surveys were calculated and t-statistics were used to assess statistical significance for the differences and calculate the 95% confidence intervals. Relative changes (% change) were calculated from survey means and standard errors were calculated using a formula derived by Fieller [29]. The same approach was carried out within each strata of household age composition (adults only, or children and adults), equivalised income tertile, and island (the main island of Tongatapu, or an outer island/island group) to examine whether post-tax changes varied. Equivalised income was calculated using the Organisation for Economic Co-operation and Development (OECD)-modified scale with a weighting of 1.0 for the first adult, 0.5 for each additional adult, and 0.3 to each child (< 14 years old). T-tests were used to assess whether the absolute changes differed between sub-group categories. A sensitivity test was carried out to remove the influence the July 2016 tax change on 2016/15 outcomes by excluding households surveyed in quarter three of both surveys (to remove the post 2016-tax period and allow for seasonal variation). Expenditure changes were then examined overall and by household income. All analyses were carried out in R using the ‘survey’ package for complex survey designs. All results adjust for survey strata, clusters and weights, to ensure results represent the national Tonga population and the true uncertainty.

## Results

Table 1 describes households in each survey population. Between surveys there were small increases in mean age of household members (2.2 years), equivalised household income (T$814, a smaller increase than inflation, which is not adjusted for here), households on the main island (2.8 percentage point (absolute) increase), mean household size (0.5 persons increase), and households that reported bottled water as their main drinking water source (6.0 percentage point increase).

### Changes in expenditure outcomes

**Fig. 1:**
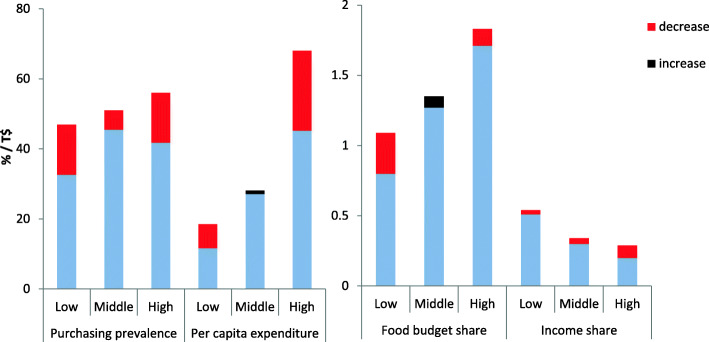
Comparison of 2009 and 2015/16 soft drink expenditure outcomes in Tonga. Notes: Household income was equivalised by household age composition using the OECD measure (see Methods) and categorised into tertiles. Expenditure was inflation adjusted. Food expenditure (used to calculate food budget share) excludes takeaway expenditures. All measures include all households. Increases and decreases are a comparison of 2015/16 outcomes after SB tax introduction with 2009 outcomes. Data source: Tonga Household Income and Expenditure Survey data, Department of Statistics, Tonga

**Fig. 2:**
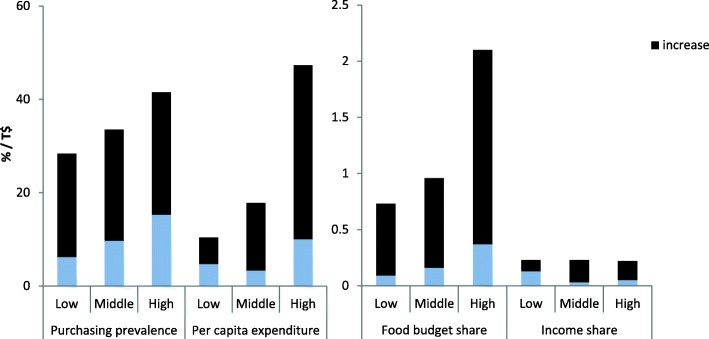
Comparison of 2009 and 2015/16 bottled water expenditure outcomes in Tonga. Notes: Household income was equivalised by household age composition using the OECD measure (see Methods) and categorised into tertiles. Expenditure was inflation adjusted. Food expenditure (used to calculate food budget share) excludes takeaway expenditures. All measures include all households. Increases and decreases are a comparison of 2015/16 outcomes after SB tax introduction with 2009 outcomes. Data source: Tonga Household Income and Expenditure Survey data, Department of Statistics, Tonga

Table 2 details the post-tax changes in beverage expenditure outcomes. There was a pattern of relative decreases in soft drink: purchasing prevalence (− 21, 95% confidence interval (CI): − 29% to − 13%), real per capita expenditure (− 22%, CI: − 44 to 3%), food budget share (− 5%, CI: − 26 to 17%), and income share (− 17%, CI: − 45 to 13%, Fig. 1). Conversely, there were large and typically significant increases in bottled water: purchasing prevalence (237%, CI: 176 to 306%), expenditure (341%, CI: 156 to 621%), food budget share (533%, CI: 231 to 906%) and income share (234%, CI: − 64 to 1072%, Fig. 2). In the same direction there were significant increases in milk purchasing: prevalence (49%, CI: 31 to 68%), expenditure (56%, CI: 21 to 94%), food budget share (94, 59 to 132%), and income share (43%, CI: 12 to 79%). The sensitivity test demonstrated similar declines in soft drink expenditure outcomes (Additional File Table S4a).

**Table 2:** Mean household beverage expenditure in Tonga before and after a sweetened-beverage tax

Outcome		2009 (95% CIs)	2015/16 (95% CIs)	Absolute difference (t-test 95% CIs)	% change (95% CI)	***p***-value
Purchasing prevalence (% households)	All beverages	72.3 (69.6 to 74.9)	80.9 (78.4 to 83.3)	8.6 (5.0 to 12.2)	11.9 (6.6 to 17.3)	< 0.001
	Soft drinks	51.3 (48.3 to 54.3)	40.4 (37.3 to 43.5)	−10.9 (− 15.2 to −6.5)	−21.2 (−28.7 to − 13.4)	< 0.001
	Milk	29.9 (27.3 to 32.4)	44.5 (40.7 to 48.4)	14.6 (10.0 to 19.3)	49.0 (31.3 to 67.8)	< 0.001
	Bottled water	10.4 (8.7 to 12.1)	35.0 (31.5 to 38.4)	24.6 (20.7 to 28.4)	236.5 (176.3 to 306.0)	< 0.001
Mean expenditure (2015/16 T$ /person/year)	All beverages	83.8 (73.6 to 94.0)	116.0 (100.6 to 131.4)	32.2 (13.7 to 50.7)	38.4 (14.4 to 64.5)	0.001
	Soft drinks	37.9 (31.1 to 44.7)	29.6 (22.5 to 36.8)	−8.2 (−18.1 to 1.6)	−21.8 (−44.1 to 3.2)	0.102
	Milk	22.7 (19.7 to 25.8)	35.4 (28.7 to 42.2)	12.7 (5.2 to 20.1)	55.7 (20.6 to 93.6)	0.001
	Bottled water	6.0 (3.4 to 8.6)	26.6 (20.9 to 32.3)	20.5 (14.3 to 26.8)	341.1 (155.7 to 621.0)	< 0.001
	All food and beverages	2500 (2310 to 2680)	2150 (2020 to 2280)	− 344 (− 573 to − 115)	−13.8 (−21.9 to − 5.2)	0.003
Beverage expenditure as a proportion of household food budget (%)	All	3.20 (2.94 to 3.45)	5.41 (4.61 to 6.21)	2.22 (1.38 to 3.06)	69.4 (41.5 to 98.3)	< 0.001
	Soft drinks	1.39 (1.24 to 1.55)	1.32 (1.07 to 1.58)	−0.07 (−0.37 to 0.22)	−5.2 (−25.7 to 16.5)	0.633
	Milk	0.87 (0.77 to 0.97)	1.68 (1.44 to 1.93)	0.82 (0.55 to 1.08)	94.2 (59.4 to 131.7)	< 0.001
	Bottled water	0.21 (0.14 to 0.28)	1.31 (0.78 to 1.85)	1.11 (0.56 to 1.65)	532.5 (230.7 to 906.0)	< 0.001
Beverage expenditure per person as a proportion of equivalised income (%)	All	0.87 (0.75 to 1.00)	1.20 (1.02 to 1.39)	0.33 (0.11 to 0.56)	38.3 (10.5 to 69.0)	0.004
	Soft drinks	0.39 (0.33 to 0.45)	0.32 (0.23 to 0.42)	−0.07 (−0.18 to 0.05)	− 17.0 (−44.5 to 12.8)	0.267
	Milk	0.23 (0.19 to 0.27)	0.33 (0.28 to 0.38)	0.10 (0.04 to 0.17)	43.2 (12.0 to 79.1)	0.002
	Water	0.07 (0.00 to 0.13)	0.23 (0.18 to 0.29)	0.16 (0.08 to 0.25)	234.3 (−63.5 to 1072.1)	< 0.001
Estimated quantity of beverages acquired (kg/person/year)	All		56.1 (46.6 to 65.5)			
	Soft drinks		10.2 (7.9 to 12.4)			
	Milk		10.3 (8.3 to 12.2)			
	Bottled water		27.5 (20.7 to 34.4)			

The 2009 expenditure findings were increased to adjust for inflation between 2009 and 2015/16 (12.4% increase, using CPI data from the Department of Statistics). *P*-values were calculated using the t-test for the difference between two means on an absolute scale. Absolute changes were expressed as a percentage (%) change by dividing the absolute change and its confidence intervals by the 2009 outcome value. The 2013 Tonga SB tax applied to soft drinks. Figures for quantity of beverages were estimated from price, available only for 2015/16.The complex survey design was taken into account in analysis to ensure findings represent the national Tongan population. Data source: Tonga Department of Statistics, Household Income and Expenditure Surveys

### Changes in expenditure by household income, household composition and island

The decreases in soft drink expenditure and increases in other beverage expenditure appeared to differ by household income per capita, age composition of the household, and island; but patterns differed between absolute and relative measures of change and some may have been due to chance (Tables 3, 4, 5 and 6). Relative changes are summarised here for international comparability.

**Table 3:** Changes in the *prevalence of households purchasing* beverages in Tonga from 2009 to 2015/16

Characteristic			Purchasing prevalence (% households)				
			2009	2015/16	Absolute difference	Relative change (%)	Diff. ***p***-value
Soft drinks (taxed in 2013)	Total	All	51.3 (48.3 to 54.3)	40.4 (37.3 to 43.5)	−10.9 (−15.2 to −6.5)	−21.2 (−28.7 to − 13.4)	< 0.001
	Age	Adults only	45.8 (41.3 to 50.4)	34.4 (28.2 to 40.6)	−11.4 (−19.1 to −3.7)	−24.9 (−40.1 to −9.0)	0.004
		Adults and children	53.2 (49.7 to 56.7)	42.8 (39.1 to 46.4)	−10.5 (−15.5 to −5.4)	−19.6 (−28.1 to − 10.8)	< 0.001
		*p-value*			0.835		
	Income tertile	Lowest	46.9 (42.0 to 51.7)	32.6 (27.2 to 38.0)	−14.3 (−21.5 to −7.0)	−30.4 (−43.6 to − 16.5)	< 0.001
		Middle	51.0 (46.8 to 55.1)	45.5 (40.7 to 50.2)	−5.5 (−11.9 to 0.8)	− 10.8 (−22.4 to 1.3)	0.087
		Highest	56.0 (51.6 to 60.3)	41.8 (37.1 to 46.5)	−14.1 (−20.6 to −7.7)	−25.3 (−35.3 to − 14.8)	< 0.001
		*p-value*			0.980		
	Island	Outer islands	35.1 (30.7 to 39.5)	23.1 (18.5 to 27.7)	−12.0 (−18.3 to −5.6)	−34.1 (−49.1 to − 18.0)	< 0.001
		Tongatapu (main island)	58.6 (54.7 to 62.4)	47.2 (43.3 to 51.2)	−11.3 (−16.9 to −5.8)	−19.4 (−27.8 to −10.6)	< 0.001
		*p-value*			0.886		
Milk	Total	All	29.9 (27.3 to 32.4)	44.5 (40.7 to 48.4)	14.6 (10.0 to 19.3)	49.0 (31.3 to 67.8)	< 0.001
	Age	Adults only	26.3 (22.1 to 30.4)	39.7 (33.8 to 45.7)	13.4 (6.2 to 20.7)	51.2 (19.9 to 86.4)	< 0.001
		Adults and children	31.1 (28.0 to 34.3)	46.4 (42.1 to 50.7)	15.2 (9.9 to 20.5)	48.9 (29.3 to 70.0)	< 0.001
		*p-value*			0.698		
	Income tertile	Lowest	22.9 (19.5 to 26.3)	36.2 (30.6 to 41.9)	13.3 (6.7 to 19.9)	58.0 (25.7 to 93.9)	< 0.001
		Middle	30.9 (26.7 to 35.1)	46.0 (40.5 to 51.4)	15.0 (8.2 to 21.9)	48.6 (23.2 to 76.9)	< 0.001
		Highest	35.8 (32.1 to 39.5)	49.6 (43.4 to 55.8)	13.8 (6.6 to 21.1)	38.7 (16.9 to 62.1)	< 0.001
		*p-value*			0.913		
	Island	Outer islands	18.8 (15.1 to 22.5)	27.3 (23.2 to 31.3)	8.5 (2.9 to 14.0)	44.9 (11.5 to 84.2)	0.003
		Tongatapu (main island)	34.8 (31.5 to 38.2)	51.3 (46.3 to 56.3)	16.4 (10.4 to 22.5)	47.2 (27.7 to 68.1)	< 0.001
		*p-value*			0.055		
Bottled water	Total	All	10.4 (8.7 to 12.1)	35.0 (31.5 to 38.4)	24.6 (20.7 to 28.4)	236.5 (176.3 to 306.0)	< 0.001
	Age	Adults only	9.4 (6.8 to 12.0)	31.9 (25.5 to 38.2)	22.5 (15.7 to 29.4)	240 (135 to 375)	< 0.001
		Adults and children	10.8 (8.7 to 12.8)	36.2 (32.6 to 39.8)	25.4 (21.3 to 29.5)	236 (169 to 316)	< 0.001
		*p-value*			0.477		
	Income tertile	Lowest	6.2 (4.1 to 8.3)	28.4 (23.5 to 33.2)	22.1 (16.8 to 27.4)	355 (203 to 563)	< 0.001
		Middle	9.7 (6.7 to 12.6)	33.5 (28.5 to 38.5)	23.8 (18.0 to 29.6)	246 (141 to 385)	< 0.001
		Highest	15.3 (12.1 to 18.4)	41.5 (36.1 to 47.0)	26.3 (20.0 to 32.6)	172 (111 to 246)	< 0.001
		*p-value*			0.324		
	Island	Outer islands	2.0 (1.0 to 3.1)	19.3 (15.6 to 23.0)	17.2 (13.4 to 21.1)	849 (399 to 1606)	< 0.001
		Tongatapu (main island)	14.2 (11.7 to 16.6)	41.2 (36.7 to 45.7)	27.0 (21.9 to 32.1)	191 (136 to 255)	< 0.001
		*p-value*			0.003		

All values are survey weighted to be representative of the national Tongan population. Absolute differences were calculated from the difference between the two means, with t-tests used to give the 95% confidence interval (CI) for the absolute difference and the *p*-value. Percentage change confidence intervals were calculated using the Fieller method [29]. The absolute difference *p*-value tests the null hypothesis that the absolute changes in outcome were the same in each sub-group, for example comparing the highest and lowest income group. The difference (Diff.) *p*-value tests for whether there was any evidence against the null hypothesis that there was no change over time between the two surveys. Source: Tonga Department of Statistics

**Table 4:** Changes in per capita *beverage expenditure* in Tonga from 2009 to 2015/16

Characteristic			Mean expenditure (2015/2016 T$/person/year)				
			2009	2015/16	Absolute difference	Relative change (%)	Diff. *p*-value
Soft drinks (taxed in 2013)	Total	All	37.9 (31.1 to 44.7)	29.6 (22.5 to 36.8)	−8.2 (−18.1 to 1.6)	−21.8 (− 44.1 to 3.2)	0.102
	Age	Adults only	67.1 (43.9 to 90.3)	47.5 (26.2 to 68.8)	−19.7 (−51.2 to 11.8)	−29.3 (− 65.7 to 16.3)	0.221
		Adults and children	27.5 (24.0 to 31.0)	22.7 (18.4 to 26.9)	−4.8 (−10.4 to 0.7)	−17.6 (−35.7 to 1.9)	0.087
		*p-value*			0.364		
	Income tertile	Lowest	18.5 (14.5 to 22.5)	11.6 (7.1 to 16.1)	−6.9 (−12.9 to − 0.9)	−37.3 (− 63.6 to −7.9)	0.024
		Middle	27.1 (23.0 to 31.2)	28.1 (17.0 to 39.2)	1.0 (−10.8 to 12.8)	3.6 (−39.0 to 48.8)	0.870
		Highest	68.0 (49.0 to 87.0)	45.2 (30.9 to 59.5)	−22.8 (−46.6 to 1.0)	−33.6 (−59.4 to − 2.2)	0.060
		*p-value*			0.203		
	Island	Outer islands	16.4 (12.7 to 20.1)	12.5 (8.8 to 16.2)	−3.9 (−9.2 to 1.4)	−23.8 (−50.6 to 7.1)	0.147
		Tongatapu (main island)	47.6 (37.8 to 57.3)	36.4 (26.5 to 46.3)	−11.2 (−25.0 to 2.7)	−23.5 (−48.0 to 4.4)	0.114
		*p-value*			0.337		
Milk	Total	All	22.7 (19.7 to 25.8)	35.4 (28.7 to 42.2)	12.7 (5.2 to 20.1)	55.7 (20.6 to 93.6)	0.001
	Age	Adults only	32.4 (24.4 to 40.3)	56.4 (39.2 to 73.5)	24.0 (5.1 to 42.9)	74.0 (10.7 to 148.4)	0.013
		Adults and children	19.3 (16.4 to 22.2)	27.2 (22.4 to 32.0)	7.9 (2.3 to 13.5)	40.8 (9.8 to 75.1)	0.006
		*p-value*			0.110		
	Income tertile	Lowest	10.8 (8.1 to 13.5)	13.9 (10.2 to 17.5)	3.0 (−1.5 to 7.6)	28.2 (− 15.3 to 80.5)	0.193
		Middle	17.5 (14.1 to 21.0)	28.1 (21.5 to 34.6)	10.5 (3.1 to 18.0)	59.9 (13.7 to 112.8)	0.006
		Highest	39.9 (32.2 to 47.5)	59.0 (43.2 to 74.8)	19.2 (1.6 to 36.7)	48.1 (1.8 to 100.0)	0.032
		*p-value*			0.082		
	Island	Outer islands	12.7 (8.6 to 16.8)	16.9 (12.6 to 21.2)	4.1 (−1.8 to 10.1)	32.5 (− 16.3 to 96.0)	0.171
		Tongatapu (main island)	27.3 (23.2 to 31.3)	42.7 (33.6 to 51.8)	15.5 (5.5 to 25.4)	56.7 (17.6 to 99.4)	0.002
		*p-value*			0.056		
Bottled water	Total	All	6.0 (3.4 to 8.6)	26.6 (20.9 to 32.3)	20.5 (14.3 to 26.8)	341.1 (155.7 to 621.0)	< 0.001
	Age	Adults only	12.3 (3.5 to 21.2)	42.8 (29.5 to 56.0)	30.4 (14.5 to 46.4)	247 (18 to 721)	< 0.001
		Adults and children	3.8 (2.7 to 4.8)	20.2 (15.5 to 25.0)	16.4 (11.6 to 21.3)	435 (257 to 658)	< 0.001
		*p-value*			0.100		
	Income tertile	Lowest	4.7 (−1.6 to 11.1)	10.4 (6.8 to 14.1)	5.7 (−1.6 to 13.0)	121 (− 964 to 6401)	0.126
		Middle	3.3 (1.4 to 5.3)	17.8 (12.4 to 23.2)	14.4 (8.7 to 20.2)	433 (134 to 963)	< 0.001
		Highest	10.0 (6.1 to 13.9)	47.3 (35.8 to 58.8)	37.3 (25.1 to 49.4)	373 (182 to 645)	< 0.001
		*p-value*			< 0.001		
	Island	Outer islands	0.4 (0.2 to 0.6)	13.8 (8.9 to 18.8)	13.5 (8.5 to 18.5)	3678 (1530 to 7300)	< 0.001
		Tongatapu (main island)	8.6 (4.8 to 12.4)	31.6 (23.9 to 39.2)	23.0 (14.5 to 31.5)	269 (107 to 512)	< 0.001
		*p-value*			0.059		

Notes: The 2009 expenditure findings were increased to adjust for inflation between 2009 and 2015/16 (12.4% increase, using CPI data from the Department of Statistics). Household expenditure was then divided by the number of people in the household. See also the footnote to Table 3

**Table 5:** Changes in beverage expenditure as a *proportion of the food budget* in Tonga from 2009 to 2015/16

Characteristic			Beverage expenditures as a proportion of food and non-alcoholic beverages expenditure (%)				
			2009	2015/16	Absolute difference	Relative change (%)	Diff. ***p***-value
Soft drinks (taxed in 2013)	Total	All	1.39 (1.24 to 1.55)	1.32 (1.07 to 1.58)	−0.07 (− 0.37 to 0.22)	−5.2 (− 25.7 to 16.5)	0.633
	Age	Adults only	1.86 (1.43 to 2.29)	1.58 (0.89 to 2.27)	−0.28 (− 1.10 to 0.53)	−15.2 (− 55.1 to 29.5)	0.497
		Adults and children	1.23 (1.10 to 1.36)	1.22 (1.02 to 1.42)	−0.01 (− 0.24 to 0.23)	−0.6 (− 19.4 to 19.3)	0.953
		*p-value*			0.525		
	Income tertile	Lowest	1.09 (0.88 to 1.29)	0.80 (0.60 to 0.99)	−0.29 (− 0.57 to − 0.01)	− 26.7 (− 47.8 to − 3.0)	0.041
		Middle	1.27 (1.05 to 1.49)	1.35 (1.02 to 1.67)	0.08 (− 0.31 to 0.47)	6.4 (−23.7 to 39.8)	0.687
		Highest	1.83 (1.49 to 2.17)	1.71 (1.12 to 2.30)	−0.12 (− 0.80 to 0.56)	−6.6 (− 41.7 to 31.8)	0.727
		*p-value*			0.652		
	Island	Outer islands	0.61 (0.49 to 0.72)	0.46 (0.35 to 0.57)	−0.14 (− 0.30 to 0.01)	− 23.9 (− 45.7 to 0.7)	0.073
		Tongatapu (main island)	1.75 (1.53 to 1.97)	1.66 (1.31 to 2.01)	−0.09 (− 0.50 to 0.33)	−5.1 (− 27.8 to 19.1)	0.676
		*p-value*			0.804		
Milk	Total	All	0.87 (0.77 to 0.97)	1.68 (1.44 to 1.93)	0.82 (0.55 to 1.08)	94.2 (59.4 to 131.7)	< 0.001
	Age	Adults only	0.81 (0.61 to 1.00)	2.02 (1.45 to 2.59)	1.21 (0.61 to 1.81)	150.2 (64.1 to 251.4)	< 0.001
		Adults and children	0.89 (0.77 to 1.01)	1.55 (1.33 to 1.78)	0.66 (0.41 to 0.92)	74.7 (41.6 to 111.2)	< 0.001
		*p-value*			0.099		
	Income tertile	Lowest	0.67 (0.51 to 0.82)	1.09 (0.85 to 1.33)	0.42 (0.14 to 0.71)	63.4 (14.8 to 121.2)	0.004
		Middle	0.85 (0.68 to 1.03)	1.58 (1.25 to 1.91)	0.73 (0.35 to 1.10)	85.1 (33.8 to 144.7)	< 0.001
		Highest	1.08 (0.89 to 1.26)	2.24 (1.73 to 2.74)	1.16 (0.62 to 1.70)	107.8 (51.3 to 170.9)	< 0.001
		*p-value*			0.018		
	Island	Outer islands	0.41 (0.30 to 0.53)	0.83 (0.57 to 1.09)	0.42 (0.14 to 0.70)	100.8 (24.4 to 193.5)	0.004
		Tongatapu (main island)	1.07 (0.93 to 1.21)	2.02 (1.70 to 2.34)	0.95 (0.60 to 1.30)	88.6 (51.5 to 128.7)	< 0.001
		*p-value*			0.020		
Bottled water	Total	All	0.21 (0.14 to 0.28)	1.31 (0.78 to 1.85)	1.11 (0.56 to 1.65)	532.5 (230.7 to 906.0)	< 0.001
	Age	Adults only	0.25 (0.13 to 0.38)	1.92 (0.45 to 3.39)	1.66 (0.19 to 3.14)	653 (49 to 1454)	0.027
		Adults and children	0.19 (0.12 to 0.26)	1.08 (0.83 to 1.33)	0.89 (0.62 to 1.15)	464 (249 to 760)	< 0.001
		*p-value*			0.309		
	Income tertile	Lowest	0.09 (0.05 to 0.14)	0.73 (0.50 to 0.95)	0.63 (0.40 to 0.86)	665 (298 to 1218)	< 0.001
		Middle	0.16 (0.06 to 0.26)	0.96 (0.70 to 1.22)	0.80 (0.52 to 1.08)	507 (159 to 1155)	< 0.001
		Highest	0.37 (0.19 to 0.55)	2.10 (0.87 to 3.33)	1.73 (0.49 to 2.97)	468 (91 to 997)	0.006
		*p-value*			0.087		
	Island	Outer islands	0.01 (0.01 to 0.02)	0.44 (0.31 to 0.56)	0.42 (0.30 to 0.55)	2811 (1188 to 5724)	< 0.001
		Tongatapu (main island)	0.29 (0.20 to 0.39)	1.66 (0.92 to 2.40)	1.37 (0.62 to 2.12)	464 (177 to 817)	< 0.001
		*p-value*			0.015		

Note: See the footnote to Table 3

**Table 6:** Changes in beverage *expenditure as a proportion of income* in Tonga from 2009 to 2015/16

Characteristic			Household beverage expenditure as a proportion of equivalised household income (%)				
			2009	2015/16	Absolute difference	Relative change (%)	Diff. ***p***-value
Soft drinks (taxed in 2013)	Total	All	0.39 (0.33 to 0.45)	0.32 (0.23 to 0.42)	−0.07 (−0.18 to 0.05)	−17.0 (−44.5 to 12.8)	0.267
	Age	Adults only	0.63 (0.43 to 0.82)	0.51 (0.19 to 0.83)	−0.12 (− 0.49 to 0.26)	−18.6 (−72.1 to 43.5)	0.544
		Adults and children	0.31 (0.26 to 0.35)	0.25 (0.20 to 0.30)	−0.05 (− 0.12 to 0.01)	− 18.0 (−36.9 to 2.9)	0.103
		*p-value*			0.752		
	Income tertile	Lowest	0.54 (0.39 to 0.70)	0.51 (0.25 to 0.76)	−0.03 (− 0.33 to 0.26)	−6.3 (−56.6 to 52.0)	0.821
		Middle	0.34 (0.25 to 0.42)	0.30 (0.17 to 0.42)	−0.04 (− 0.19 to 0.11)	−11.4 (−51.9 to 35.2)	0.617
		Highest	0.29 (0.23 to 0.36)	0.20 (0.16 to 0.24)	−0.09 (− 0.17 to − 0.02)	−31.5 (− 50.4 to −9.1)	0.017
		*p-value*			0.712		
	Island	Outer islands	0.19 (0.14 to 0.24)	0.17 (0.11 to 0.23)	−0.02 (− 0.09 to 0.06)	−8.9 (−45.2 to 34.3)	0.668
		Tongatapu (main island)	0.48 (0.39 to 0.57)	0.38 (0.25 to 0.52)	−0.10 (− 0.26 to 0.06)	−20.2 (−50.7 to 13.1)	0.240
		*p-value*			0.379		
Milk	Total	All	0.23 (0.19 to 0.27)	0.33 (0.28 to 0.38)	0.10 (0.04 to 0.17)	43.2 (12.0 to 79.1)	0.002
	Age	Adults only	0.32 (0.19 to 0.46)	0.43 (0.30 to 0.55)	0.10 (−0.08 to 0.29)	32.1 (−26.4 to 116.3)	0.264
		Adults and children	0.20 (0.17 to 0.23)	0.30 (0.25 to 0.34)	0.10 (0.04 to 0.15)	47.9 (17.3 to 82.1)	0.001
		*p-value*			0.937		
	Income tertile	Lowest	0.33 (0.22 to 0.44)	0.42 (0.31 to 0.54)	0.10 (−0.06 to 0.25)	28.9 (−19.6 to 92.3)	0.229
		Middle	0.20 (0.16 to 0.24)	0.30 (0.23 to 0.37)	0.10 (0.02 to 0.18)	47.9 (5.1 to 97.6)	0.018
		Highest	0.17 (0.14 to 0.20)	0.29 (0.23 to 0.36)	0.13 (0.05 to 0.20)	75.7 (27.1 to 130.3)	0.001
		*p-value*			0.716		
	Island	Outer islands	0.14 (0.08 to 0.20)	0.21 (0.17 to 0.25)	0.07 (0.00 to 0.14)	48.7 (−11.8 to 139.3)	0.065
		Tongatapu (main island)	0.27 (0.22 to 0.33)	0.38 (0.31 to 0.45)	0.11 (0.02 to 0.19)	39.4 (5.2 to 79.2)	0.014
		*p-value*			0.498		
Bottled water	Total	All	0.07 (0.00 to 0.13)	0.23 (0.18 to 0.29)	0.16 (0.08 to 0.25)	234.3 (−63.5 to 1072.1)	< 0.001
	Age	Adults only	0.16 (−0.08 to 0.40)	0.29 (0.18 to 0.41)	0.13 (− 0.14 to 0.40)	80 (644 to − 4542)	0.336
		Adults and children	0.04 (0.03 to 0.05)	0.21 (0.16 to 0.25)	0.17 (0.12 to 0.22)	479 (284 to 723)	< 0.001
		*p-value*			0.775		
	Income tertile	Lowest	0.13 (−0.06 to 0.32)	0.23 (0.15 to 0.32)	0.11 (−0.10 to 0.31)	83 (666 to − 4527)	0.311
		Middle	0.03 (0.02 to 0.05)	0.23 (0.12 to 0.35)	0.20 (0.09 to 0.31)	603 (200 to 1166)	< 0.001
		Highest	0.05 (0.03 to 0.06)	0.22 (0.17 to 0.28)	0.18 (0.12 to 0.23)	397 (217 to 637)	< 0.001
		*p-value*			0.495		
	Island	Outer islands	0.01 (0.00 to 0.01)	0.13 (0.09 to 0.16)	0.12 (0.08 to 0.16)	1962 (− 282 to 9346)	< 0.001
		Tongatapu (main island)	0.10 (0.00 to 0.19)	0.27 (0.20 to 0.35)	0.17 (0.05 to 0.29)	179 (−81 to 939)	0.004
		*p-value*			0.397		

Note: See the footnote to Table 3

The soft drink declines in expenditure outcomes showed a pattern of being somewhat greater in low-income than high-income households. This was for purchasing prevalence (low-income: − 30% and high-income:-25%, *p* = 0.98), per capita expenditure (− 37% and − 34%, *p* = 0.20) and food budget share (− 27% and − 7%, *p* = 0.65), but not income share (− 6% and − 32%, *p* = 0.71) (Fig. 1, Tables 3, 4, 5 and 6). For middle-income households there was a smaller decline in soft drink purchasing prevalence, and indeed small increases in soft drink expenditure, food budget share and income share. Declines were greater in adult only households compared to households with children for soft drink purchasing prevalence, average expenditure and food budget share (income share declines were similar in both). Declines in purchasing prevalence and income share were greater in Tongatapu households; whereas declines in food budget share appeared to be greater in outer island households (expenditure declines were similar by island type).

The increases in bottled water showed a pattern of being greater in low-income than high-income households. This was for purchasing prevalence (355 and 172% respectively, *p* = 0.32) and food budget share (665 and 168%, *p* = 0.09) but increases were greater in high-income households for per capita expenditure (121 and 373%, *p* < 0.01), and income share (83 and 397%, *p* = 0.50) (Fig. 2, Tables 3, 4, 5 and 6). Adult only households appeared to have greater increases in bottled water food budget share, and households with children had greater increases in bottled water per capita expenditure and income share (with purchasing prevalence similar in both). Outer island households had greater bottled water expenditure increases for all outcomes compared to households in Tongatapu. There was a mixed pattern across milk expenditure outcomes by household income.

In the sensitivity analysis with post-2016 tax effects removed, the relative changes in bottled water expenditure showed similar patterns by household income but soft drinks expenditure declines were typically greater in high-income than low-income households (Additional File Table 4b). When the quarter after the July 2016 tax increase was compared with the three quarters before; low-income households showed a pattern of greater declines than high-income households in soft drink purchasing prevalence, mean expenditure, food budget share and income share, but lower declines in beverage quantity (not significant, Additional File Tables S5–6).

## Discussion

### Main findings

#### Taxed beverages

In 2015/16, there were 5 to 22% decreases in all soft drink expenditure outcomes but not all changes were at a statistically significant level. These decreases were robust to sensitivity analysis that excluded the effects of the 2016 tax increase. They were typically larger than the previously reported decline in SB import volumes in the year after the 2013 SB tax (−10% [18]). Also the reported decreases in expenditure, as a proportion of the 27% tax increase in Tonga, were typically less than the impact of SSB taxes on purchasing or dietary intake in a meta-analysis (average of a 10% decline for a 10% tax) [1] and that reported by recent studies from Berkeley, US [30] and Philadelphia, US [31]. But they were more similar to the proportional impact of SSB taxes on sales in Barbados [32] and Seattle, US [33].

We could not compare volume changes over time, although they can be approximated using Tonga Department of Statistics store price data. From 2010 to 2016 the price of an indicator beverage of 600 ml Coca-Cola increased from T$2.03 to T$2.63 (Tonga Department of Statistics). If we assumed that this was the average price per litre for all soft drinks, then the average decrease in soft drink expenditure volume would have been from 10.0 L to 6.8 L/person/year; which is a greater decline than the expenditure decline (32% vs 22%).

There was a pattern of greater soft drink expenditure declines in low-income households compared to high-income ones, but these results were not statistically significant or robust to sensitivity analysis where the effects of the 2016 tax increase were removed. Despite this, the pattern of greater low-income decline in expenditure is consistent with results from other jurisdictions such as Mexico [7, 34]. Low-income households may respond more than high-income households, because of greater price sensitivity, and as a result of a newly introduced volumetric tax design (per L) which disproportionately increased prices of cheaper taxed products [4].

#### Untaxed beverages

There were large increases in all bottled water and milk expenditure measures. Substitution to water and other untaxed beverages has also been found by other studies after the introduction of SB taxes [1], but the magnitude of the bottled water expenditure increases seen here for Tonga were much greater. Findings were consistent with large post-tax increases in the levels of water bottling in Tonga [18] and a 2017 household survey that indicated that 23% of respondents switched to bottled water after the SB tax in Tonga [17]. Although positive for health compared to soft drinks, increased purchasing of bottled water might be problematic compared to using rain water that is safe for drinking. This is because both soft drinks and bottled water contribute to litter, use up limited landfill space, and can increase net costs for households. Furthermore, littered containers can provide a breeding site for mosquito vectors of disease [35] such as dengue fever (which causes occasional outbreaks in Tonga).

There was a mixed pattern of bottled water increases by income. Relative increases in per capita expenditure were greater in high-income households (statistically significant), households with children and in the outer islands (although adult only and main island households had the greatest absolute increases in expenditure). Findings suggest that the rapidly growing water bottling industry on the main island has particularly benefited those who can access and afford it. High-income households were fivefold more likely to depend on bottled water as their main drinking water source than low-income households in 2015/16. The drivers of increased bottled water expenditure are likely to do with greater retail availability; however it is unclear why bottled water is increasingly preferred over other water sources, and whether this is affected by concerns about drinking water safety or palatability when it is from household tanks.

### Study strengths and limitations

A major strength of this study was the ability to disaggregate changes in expenditure by household age composition, income and island, using two nationally representative surveys and targeting all spending, gifts and food received. Statistical power in these comparisons was limited by survey size. However, it remained important to examine disaggregated patterns because ‘non-significant’ findings can still be useful [36], particularly in domains of major health impact. The study focussed on household reports of soft drink expenditure consumed in the home, bottled water and milk. Juice drinks, cordial and flavoured milk could not be included due to changes in coding between the surveys. Also, there were no data on tap water and beverages consumed outside the home as takeaways, in restaurants or at school. Nevertheless, the effects of takeaway expenditures may be limited given the small 2% increase in reported takeaway food and beverage expenditure during the study period, and the greater increase in high-income households. Expenditure surveys have been found to underestimate expenditure compared to nutrition focussed studies [37], however there was no suggestion that any under-reporting increased over time.

This was an observational study and therefore results are also likely to have been affected by other changes over time such as (eg, macroeconomic changes from commodity price fluctuations or tourism flows). There were some minor changes in household characteristics between surveys, but key expert interviews did not identify any other major policies or programmes that might have influenced SB intake during the study period. Data were not available to compare trends in Tonga with those from a comparable jurisdiction or with pre-existing trends. Furthermore, there was a time lag issue with the 2009 pre-tax change data being collected 4 years before the 2013 tax was implemented.

The lack of price data in this analysis was a limitation. In interpreting expenditure trends, decreasing average soft drink prices (or substitution to cheaper beverages) may have contributed to reduced soft drink expenditure without impacting on the volume consumed [4] and trends may differ by household income. Although, as noted above, the price of an indicator soft drink increased over time, increasing volumes of cheaper SBs imported from Malaysia (Additional File Figure A) may have been more likely to be purchased by low-income households. In 2015/16, the average soft drink price for beverages purchased in low-income households was slightly lower at T$2.60/L than that for high-income households at T$2.90/L (Table 4 and S7), but it is unclear whether this changed since 2009 so any impact on trends by household income are unknown.

### Potential implications

SB taxes appeared to be associated with some pro-equity effects in Tonga, particularly in the reduction of soft drink expenditure. However, the equity impacts of SB taxes may be further enhanced through improved policy design. SB taxes should include all SBs to prevent substitution to beverages that might be attractive as cheaper alternatives (such as juice drinks, sachet drinks, cheap imported soft drinks and locally-produced beverages). The Tongan SB excise was broadened to include sugar-sweetened juice and sachet drinks in 2017, but the equity impacts of this change are as yet unknown. Volumetric (per litre) [3] and more recently nutritional taxes (per sugar content) [38], have been recommended to improve the effectiveness of SB taxes, and may increase the relative response from low-income households and purchasers of cheaper beverages [3]. Revenue from the SB tax can be invested back into health or wellbeing [6], for example to ensure availability of safe drinking water (safety of rain water collection tanks or a treated reticulated drinking water supply) for the whole population. This is likely to support healthy beverage substitution and greater equity of SB tax effects; and is particularly important for households that may not be able to afford bottled water, households with children, and in the outer islands. Planned monitoring designed to assess the equity impacts of SB tax changes can inform ongoing health and equity improvements to the policy design. Further research into the socioeconomic patterns of beverage consumption changes in response to SB taxes is needed to further understand equity impacts of SB taxes in different settings.

## Conclusions

The sweetened-beverage tax in Tonga was associated with reduced soft drink purchasing and increased bottled water expenditure. Low-income households appeared to benefit from greater relative declines in soft drink expenditure but high-income households appeared to have greater increases in bottled water expenditure.

## Supplementary Information

**Additional file 1.** Further methods, results and discussion. Additional information on the methods, results and discussion from this research study including sensitivity analyses. Please see this file for all additional tables

## Data Availability

The data that support the findings of this study are available from Pacific Community but restrictions apply to the availability of these data, which were used under license for the current study, and so are not publicly available. Data are however available from the Pacific Community upon reasonable request and with permission of Tonga Department of Statistics.

## Abbreviations

CI: Confidence interval
HIES: Household income and expenditure survey
NCD: NON-communicable disease
OECD: Organisation for Economic Co-operation and Development
SB: sweetened beverage
SEP: Socioeconomic position
SSB: Sugar-sweetened beverage
