# Supplementary material for: Equity of expenditure changes associated with a sweetened-beverage tax in Tonga: repeated cross-sectional household surveys
Source: BMC Public Health. 2021 Jan 18;21:149. doi: 10.1186/s12889-020-10139-z (PMC7812720; doi:10.1186/s12889-020-10139-z)
Supplement: Supplementary file 1 — Additional file 1. Further methods, results and discussion. Additional information on the methods, results and discussion from this research study including sensitivity analyses. Please see this file for all additional tables [file 12889_2020_10139_MOESM1_ESM.docx]

# Additional File 1: Further methods, results and discussion

Andrea Teng, Bertrand Buffière, Murat Genç, Telekaki Latavao, Viliami Puloka, Louise Signal, Nick Wilson. Equity of expenditure changes associated with a sweetened-beverage tax in Tonga: Repeated cross-sectional household surveys.

Contents

[Further methods 1](#_Toc34920299)

[Study analyses 1](#_Toc34920300)

[Sensitivity test 5](#_Toc34920301)

[Additional analyses 5](#_Toc34920302)

[Further results 6](#_Toc34920303)

[Sensitivity test 6](#_Toc34920304)

[Expenditure changes within the 2015/16 survey 8](#_Toc34920305)

[Expenditure volume patterns in 2015/16 10](#_Toc34920306)

[Origin of imported beverages 11](#_Toc34920307)

[Further discussion 12](#_Toc34920308)

[Further strengths and limitations of this study 13](#_Toc34920309)

[References 13](#_Toc34920310)

## Further methods

### Study analyses

Tables A to C provide more information about the HIES survey designs, beverage category coding and variable derivation respectively.

**Supplementary Table S1: Household income and expenditure survey designs in Tonga**

| **Tonga** | **2009** | **2015/16** |
| --- | --- | --- |
| **SB taxes** | 15% tariff only | After the 2013 SB excise; and 9 months before and 3 months after the 2016 SB tax increase from T$0.50 to T$1.00/L |
| **Author and purpose** | Tonga Statistics Department, technical assistance from SPC  To support CPI, national accounts and measure income  Data coding and data entry at Statistics Department, some delays. Cleaning. | Pacific Community (SPC) and the Statistics Department  Support CPI, national accounts, and measure poverty  Telekaki Latavao, Senior Statistician, Tonga Department of Statistics (HIES Coordinator)  Dr Haberkon, the former Manager, Statistics and Demography Program of the SPC  Dr ‘Ofa Ketu’u (SPC)  In round data entry. Cleaning. |
| **Size and scope** | 1983 households (10%)  All island divisions in each quarter, except for Ono Niua  Tongatapu (urban and rural), Vava’u, Ha’apai, ‘Eua, and Onga Niua | 1803 households  10,319 individuals (10% of the population)  Tongatapu (urban and rural), Vava’u, Ha’apai, ‘Eua, and Onga Niua |
| **Timing** | One phase each quarter | 16 rounds over 12 months (4 rounds per quarter) from October 2015 to October 2016 |
| **Survey schedules** | Resolution 1 from the Seventeenth International Conference of Labour Statisticians  • Household Questionnaire – focusing on large irregular expenditures  • Individual Questionnaire (Pt 1) – focusing on income  • Individual Questionnaire (Pt 2) – focusing on education, health and labour force  • Two 1-week Diaries – focusing on small regular expenditures and subsistence activity | Pacific standard HIES methodology 2011  New questions on deprivation  The use of a common questionnaire:  • 4 modules to collect socio-demographic information, and expenditure (household and individual) and income; and  • a two-week diary to collect daily expenditure, gifts received and home produced items. |
| **Sampling** | STRATA  6 geographical areas, oversampling from the smaller islands to improve statistical power  Excludes extreme remote areas (3.5% population)  CLUSTERS  Two stage, probability proportional to size to identify census blocks, then sampled 12 households sampled randomly within each census block  Every 4^th^ census block allocated to a different quarter.  2064 households were selected  pcb = CB number (census block, included 12 households) | STRATA  6 geographical areas, oversampling from the smaller islands to improve statistical power  Excludes extreme remote areas (3.3% population)  CLUSTERS  Two stage sampling, probability proportional to size (number of households) to identify census blocks, then randomly sampled 18 households  1824 households sampled  block_code = Block number (census block, each included 12 households, found in the cover dataset) |
| **Response rates** | 96.1% | 98.8%  Usual residents  (But additional 50% sample of households were invited if other households were absent) |
| **Food expenditure** | Food and non-alcoholic beverages were main expenditure (51%) | Food and non-alcoholic beverages were main expenditure (37%)  Half of expenditure came from one-quarter of households. |
| **Reported challenges** | Usual under reporting and misunderstanding of questions. Modification to Ha’apai sample when ferry sunk with lives lost. | Misinterpretation, fatigue and under reporting, incomplete disclosure eg income and expenditure on alcohol and tobacco, English language challenges (3/4 diaries were in Tongan), couldn’t interview HH members abroad |
| **Household variables** | Region/weights/rurality  Main source of drinking water; “What is the main source of drinking water your household uses?” | Region/weights/rurality, drinking water source, water bill expenditure  Deprivation – multiple questions, income |
| **Individual variables** | Relationship to head, sex, age, ethnicity, income employment status  No BMI or useful health measures.  Highest education level.  Employment  Income | Relationship to head, sex, age, ethnicity, income employment status  Health: measured BMI and self-reported: diabetes, cancer, heart disease, hypertension etc  Deprivation variables: inability to afford shoes, two meals a day, discretionary spending, clothes for special occasions, replace worn out clothes, meal with friends, presents for friends, visit friends in hospital, safe public transport, meat/fish on Sundays, fresh fruits once a day.  Income |
| **Beverage detailed categories** | Only available as expenditure, no quantity/weight  Labels (most disaggregated):  Milk ( Long-Life)  Process Drink (ice pop, etc)  Tea, Tea Bags & substitutes  Cocoa, Drinking Chocolate  Milo  Coffee  'Otai  Fruit Drinks (Powdered, Crystallised, Canned)  Water, Party Ice  Cordial Concentrates (Powdered-liquid)  Soft Drinks (Still) | Expenditure and quantity (weight )  Labels (specific sub-types were grouped into these categories)  Cocoa  Coffee  Dairy Milk  Fruit Juices  Milk-Based Desserts And Beverages  Mineral Or Spring Waters; All Drinking Water Sold In Containers  Other Non-Alcoholic Beverages  Soft Drinks  Syrups And Concentrates For The Preparation Of Beverages  Tea  Vegetable Juices |

Source: Summarised from (1) Kingdom of Tonga Statistics Department. Tonga Household Income and Expenditure Survey 2009. 2010; and (2) Kingdom of Tonga Statistics Department, Pacific Community. Tonga Household Income and Expenditure Survey 2015/2016. Noumea, New Caledonia October 2017.

**Supplementary Table S2: Comparability of beverage categories used to code diary expenditures in the Tonga 2009 and 2015 household expenditure surveys**

| **Study categories** | **HES 2009 categories** | **HES 2015 categories** |
| --- | --- | --- |
| Soft drinks | 012205 Soft Drinks (Still) | 12207201 Coca Cola / Kokakola  12207202 Fanta / Fenita  12207203 Fiz / Fisi  12207204 Pepsi Cola / Pepisi Kola  12207205 Seven-Up / Seveni 'Apu  12207206 Sprite / Sipulaiti  12207207 Other Sodas N E S / Ngaahi Sota Kehe  12207208 Soft Drinks Without Any Detail / Inu Sota Ikai Ha Fakaikiiki |
| Water | 012203 Water, Party Ice | 12207101 Bottled Water (With or Without Gas) / Hina Vai ('Iai Pe 'Ikai Ha Kasa) |
| Milk | 011404 Milk (Long-Life) | 11402601 Diary Liquid Milk (Long Life, Fresh, Pasteurised….) In Carton Or Bottle / Hu'Akau Vai - Katuni Pe Hina |
| Juice/other excluded (includes fruit juice, cordial concentrates, powdered drink sachets, flavoured milk, and other processed drinks) | 012201 'Otai | 12207306 Local Juice (Otai …) / Otai |
|  | 012202 Fruit Drinks (Powdered, Crystallised, Canned)  012204 Cordial Concentrates (Powdered-liquid) | 12207301 Lemon Juice, Lemonade / Inu Lemani  12207302 Mango Juice / Inu Mango  12207303 Orange Juice / Inu Moli  12207304 Pineapple Juice / Inu Faina  12207305 Tropical Mix Juice / Inu Fo'I'Akau Kehekehe  12207307 Other Fruit Juice N E S / Inu Fua'I'Akau Kehe 'Ikai 'Asi He Lisi  12207308 Fruit Juice Without Any Detail / Inu Fua'I'Akau 'Ikai Fakaikiiki  12207401 Tomato Juice / Inu Temata  12207402 Other Vegetable Juice N E S / Inu Vesitapolo Kehe 'Ikai 'Asi He Lisi  12207501 Cordial, Refresh Eg Sunquick / Inu Kotiolo  12207503 Nutri C / Nituliki (powdered fruit drink)  12207504 Staminade / Sitamina (sports drink powder) |
|  | 011902 Process Drink (ice pop, etc) | 12207505 Process Drink-Zab Drink / Inu Seepi |
|  |  | 11403302 Flavoured Milk (Chocolate Milk, Strawberry Milk Flavoured…) / Huakau Feleiva (Sokoleti, Situloapeli) (included because flavoured milk may have been included in 2009 in the process drink category) |
| Other excluded beverages | 012101 Tea, Tea Bags & substitutes | 12106801 Tea Bags / Ti  12106802 Plant Products For Infusions / Ngaahi Me'A Kehe  12106803 Other Tea Or Infusions N E S / Ti Kehe |
|  | 012102 Cocoa, Drinking Chocolate | 12106901 Cocoa  12107001 Chocolate Based Powder (Excludes Chocolate In Bars, Boxes And Cocoa-Based Dessert Preparations (01.1.8)) / Pauta Sokoleti |
|  | 012103 Milo | 12107002 Milo / Mailo |
|  | 012104 Coffee | 12106701 Coffee Beans / Piini Kofi  12106702 Instant Coffee / Kofi  12106703 Grounded Coffee / Kofi Fefeka  12106704 Other Coffee N E S / Kofi 'Ikai 'Asi He Lisi  12106705 Coffee Without Any Details / Kofi 'Ikai Ha Fakaikiiki |
|  |  | 12107003 Ovaltine / Ovolotini |
|  |  | 11403301 Custard, Milkshakes Etc / Kasitati, Milikiseiki |
| Bulk water costs | 044101 Water connection fee | 44112002 Water Connection Fees |
|  | 044102 Water – Piped | 44112001 Water Bill, Water Rates |
|  |  | 44112003 Water Truck |
|  |  | 44112004 Other Fee Related To Water Supply |
| Takeaway foods | 111101 Meals Consumed In Restaurants and Grill Rooms  111102 Meals Consumed in Hotels And Motels  111103 Food Consumed on Club Premises  111104 Food and Beverages Consumed At Outdoor Gatherings  111105 Food and Beverages Consumed in Eating Places N.E.S.  111106 Fried Fish (with or without Accompaniments)  111107 Fried Sausages, Hot Dogs, Patties (With or Without Accompaniments)  111108 Fried Chicken (With or Without Accompaniments)  111109 Fried Chips Purchased Separately  111110 Fried Foods  111111 Pies (Meat, Fruit)  111112 Hamburgers, Egg-Burgers, etc  111113 Savouries, Sausage Rolls  111114 Pizzas  111115 Chinese Food, Spring Rolls  111116 Sandwiches (Fresh, Toasted) Buttered Bread  111117 Filled Bread Rolls  111118 Salads (Vegetable, Fruit)  111119 Cakes, buns  111120 Toasted Bread  111121 Faikakai  111122 Take-Away Foods N.E.S.  111123 Take-Aways N.O.D  111124 restaurant and take away food overseas  111201 Food Consumed in Work And School Cafeterias | 111126501 Take Away Ice Cream  111126502 Take Away Pizza  111126503 Cooked Poultry, Chicken Take Away  111126504 Take Away Plate, Take Away Food (Chinese Food, Chicken, Barbecue, Cassava, Fish ….)  111126505 Take Away Plate Local Food (Faikai, Lu, Ngou'A, Vailesi, Faikakai …)  111126506 Take Away Burger, Sandwich…  111126507 Plate Of Food Without Any Detail  111126508 Lunch Money No Detail  111226701 School Restaurant |

**Supplementary Table S3: Derivation and description of study variables**

|  | **Variable name** | **Description and derivation** |
| --- | --- | --- |
| Purchasing outcomes | Purchasing prevalence | Proportion of households that reported one or more purchases of a beverage. |
|  | Expenditure per person | Household expenditure on a beverage per household member; measured in current T$ per year and 2009 expenditures were adjusted for inflation to represent the 2015/16 prices. |
|  | Beverage expenditure as a proportion of food expenditure (food budget share) | Total household expenditure on a beverage as a proportion of total household food and non-alcoholic beverage expenditure; measured as a percentage at the household level. |
|  | Expenditure per person as a proportion of equivalised household income (income share) | Total household expenditure per person as a proportion of equivalised household income. Calculated from equivalised income and per person expenditure with no adjustment for inflation. |
|  | Quantity per person | Average acquired quantity of a beverage per person in each household, measured in kg per year. This figure is estimated from price, using the median price per gram in the beverage category or local market sourced prices per gram. This variable was only available for 2015/16. |
| Stratification variables | Equivalised income tertiles | Tertiles of equivalised household income per person. The study sample was split into three even sized groups based on equivalised household income (see below). No allowance was made for survey weighting in producing these tertiles. |
|  | Age composition | Categorisation of households into adults (15+) only, or adults and children (0-14yo) only |
|  | Obesity | Households were categorised into those where all adults were obese, all adults were not obese and those where there was a mix of both. BMI was calculated from height and weight measures for adults 15+yos. In the data cleaning process some of these measures were imputed. Obesity was defined as BMI greater than or equal to 30+kg/m^2^. |
|  | Main drinking water source | Main drinking water source (categorical variable) 2015/16 only   - Household tank or another household, community or church water supply, or other (public piped or unprotected dug well). ‘Other’ was a very small category. - Bottled water |
|  | Island | In Tonga there were 6 official regions used for stratification of the analysis and 5 islands. The two Tongatapu regions were considered as one region because they were both on the main island. The outer islands were grouped together. A greater availability of beverages on the main island was expected as a result of the main port. |
| Other summary measures | Equivalised income | Total household income in each household divided by the household composition weighting scale. This measure used the OECD-modified scale with a weighting of 1.0 for the first adult, 0.5 for each additional adult, and 0.3 to each child (<14 years old). |
|  | Mean age | Mean age in each household |
|  | Mean obesity | Mean level of adult obesity in each household |
| Covariates and potential confounders | Quarter | Quarter of the year in which expenditure was recorded. |
|  | Household size | Number of persons from each household with data (consistent method between surveys) |
|  | Highest qualification | Highest qualification; either (i) Bachelor, post-graduate, (ii) Certificate, diploma, (iii) Secondary school certificate or (iv) no qualification. Categorical variable. These categories were available specifically for 2015/16. In 2009 highest level of complete education recorded as primary and kindergarten were coded as ‘no qualification’, secondary 1 and 2 were coded as ‘secondary school certificate’, university was coded as ‘bachelor, post-graduate’ and technical/vocational was coded as ‘certificate, diploma’. |

### Sensitivity test

A sensitivity test was carried out to examine the influence the 2016 tax change on 2015/16 expenditures. This was done by excluding households surveyed in the quarter three in the 2009 and 2015/16 surveys. Study outcome changes are reported overall and by household income, and then compared with the primary study results.

### Additional analyses

First, analysis was done to examine the expenditure changes within the 2015/16 survey before and after the 2016 tax change. The 2015/16 survey spanned a year and encompassed the July 2016 tax increase at the start of the final quarter of data gathering. Expenditure outcomes were therefore examined before and after 1 July 2016, within the same survey. Findings for all expenditure outcomes and all beverages were presented overall and by per capita household income tertile (Tables S5 and S6). T-tests were used to assess the differences in sub-group before-after SB tax changes. A major limitation of this analysis is that we could not account for seasonal variation and therefore it was not used as a main analysis.

In a second analysis, beverage quantities from the 2015/16 survey were examined (Table F) including an examination of heterogeneity by household age composition (15+ year old adults only, or adults and children), per capita household income tertile, island, obesity composition (all 15+ year olds were obese, no 15+ year olds were obese, or mixed), drinking water source (bottled water or other which mainly included household or community tank water supply), annual quarter and educational level of the household head. Linear regression was used to test whether there was evidence of a trend in beverage quantities across these binary and ordinal categorical variables. In the expenditure diaries, quantity was either recorded directly (rare) or as a number of bottles (24%), boxes (23%), cans (14%) or packs (14%). If the latter was the case, then quantity was estimated using price indices (median price per gram) from collected data or local market prices as investigated by the Statistics Office. These data were used to estimate price (T$/L) of purchased beverage expenditures by household income tertile.

Three, UN Comtrade data^(1)^ were also used to describe export trends of sweetened beverages (HS 2202) to Tonga by country of origin (Figure S1), as reported by the exporting country. These trends were of interest given their potential impact on beverage prices.

## Further results

### Sensitivity test

Supplementary Tables S4a-S4b present results with removal of quarter 3 data from the 2009 and 2015/16 surveys to enable a comparison of expenditure changes over time, without the effects of the doubling of the SB excise in 2016.

**Supplementary Table S4a: Sensitivity test mean household beverage expenditure in Tonga before and after the sweetened-beverage tax excluding third quarters (Table 2 without Q3)**

| **Outcome** |  | **2009** | **2015/16** | **Absolute difference** | **% change** | **p-value** |
| --- | --- | --- | --- | --- | --- | --- |
|  |  | (95% CIs) | (95% CIs) | (t-test 95% CIs) | (Kohavi 95% CI) |  |
| Purchasing prevalence  (% households) | All beverages | 75.0 (72.3 to 77.7) | 83.1 (80.2 to 86.0) | 8.1 (4.1 to 12.1) | 10.8 (5.3 to 16.4) | <0.001 |
|  | Soft drinks | 53.3 (50.1 to 56.5) | 42.5 (38.8 to 46.1) | -10.9 (-15.7 to -6.0) | -20.4 (-28.5 to -11.9) | <0.001 |
|  | Milk | 31.7 (28.7 to 34.7) | 46.1 (41.6 to 50.6) | 14.4 (9.0 to 19.8) | 45.4 (26.2 to 65.9) | <0.001 |
|  | Water | 10.8 (8.7 to 12.8) | 34.9 (30.8 to 39.1) | 24.2 (19.6 to 28.8) | 224.8 (157.2 to 304.7) | <0.001 |
| Mean expenditure (2015/16 T$ /person/year) | All beverages | 89.1 (77.0 to 101.2) | 127.7 (108.6 to 146.8) | 38.6 (16.0 to 61.3) | 43.3 (15.6 to 73.9) | 0.001 |
|  | Soft drinks | 40.8 (32.3 to 49.3) | 32.8 (23.6 to 42.0) | -8.0 (-20.5 to 4.5) | -19.6 (-46.1 to 10.5) | 0.210 |
|  | Milk | 22.9 (19.5 to 26.4) | 39.2 (30.7 to 47.7) | 16.3 (7.0 to 25.5) | 70.9 (27.4 to 118.3) | 0.001 |
|  | Water | 6.7 (3.3 to 10.2) | 28.3 (21.2 to 35.5) | 21.6 (13.7 to 29.5) | 320.2 (117.0 to 650.0) | <0.001 |
|  | All food and beverages | 2,620 (2,390 to 2,860) | 2,200 (2,050 to 2,360) | -421 (-703 to -139) | -16.0 (-25.3 to -6.1) | 0.003 |
| Beverage expenditure as a proportion of household food budget (%) | All | 3.33 (3.04 to 3.62) | 5.83 (4.81 to 6.86) | 2.51 (1.44 to 3.58) | 75.4 (41.6 to 110.6) | <0.001 |
|  | Soft drinks | 1.47 (1.29 to 1.66) | 1.44 (1.12 to 1.77) | -0.03 (-0.40 to 0.34) | -2.0 (-26.5 to 24.1) | 0.879 |
|  | Milk | 0.86 (0.75 to 0.97) | 1.80 (1.49 to 2.10) | 0.94 (0.62 to 1.26) | 109.6 (66.9 to 155.9) | <0.001 |
|  | Water | 0.22 (0.14 to 0.30) | 1.41 (0.70 to 2.12) | 1.19 (0.48 to 1.91) | 543.3 (178.1 to 1,007.7) | 0.001 |
| Beverage expenditure per person as a proportion of equivalised income (%) | All | 0.93 (0.78 to 1.09) | 1.32 (1.08 to 1.55) | 0.38 (0.10 to 0.66) | 40.9 (8.5 to 77.1) | 0.008 |
|  | Soft drinks | 0.42 (0.35 to 0.50) | 0.36 (0.23 to 0.49) | -0.06 (-0.21 to 0.09) | -14.3 (-46.9 to 21.2) | 0.425 |
|  | Milk | 0.23 (0.19 to 0.28) | 0.36 (0.30 to 0.43) | 0.13 (0.05 to 0.21) | 56.2 (18.0 to 100.9) | 0.001 |
|  | Water | 0.08 (-0.01 to 0.17) | 0.24 (0.17 to 0.30) | 0.16 (0.05 to 0.27) | 198.9 (-143.4 to 1,365.0) | 0.004 |

Notes: The 2013 Tonga SB tax applied to soft drinks. P-values were calculated using the t-test for the difference between two means on an absolute scale, testing for whether there was any difference in expenditure outcomes over time. Absolute changes were expressed as a percentage (%) change by dividing the absolute change and its confidence intervals by the 2009 outcome value. We cannot rule out seasonal impacts on these results. The 2009 expenditure findings were increased to adjust for inflation between 2009 and 2015/16 (12.4% increase, using CPI data from the Department of Statistics). The complex survey design was taken into account in analysis to ensure findings represent the national Tongan population. Data source: Tonga Department of Statistics, Household Income and Expenditure Surveys.

**Supplementary Table S4b:** **Sensitivity test changes in expenditure outcomes beverages in Tonga from 2009 to 2015/16 excluding third quarters (similar to Table 3 without Q3)**

|  |  | **Absolute difference by per capita household income tertile**  **(95% CI)** | | |  | **Percentage change**  **(Kohavi 95% CI)** | | |
| --- | --- | --- | --- | --- | --- | --- | --- | --- |
| **Outcomes** | Income tertile | Low | Middle | High | *p-value* | Low | Middle | High |
| Purchasing prevalence  (% households) | Soft drinks | -11.0 (-19.0 to -3.0) | -7.3 (-14.5 to 0.0) | -15.1 (-22.4 to -7.9) | 0.447 | -23.4 (-38.4 to -7.4) | -13.8 (-26.4 to -0.4) | -25.2 (-35.9 to -14.1) |
|  | Milk | 9.3 (2.3 to 16.3) | 13.9 (5.6 to 22.2) | 18.1 (9.6 to 26.5) | 0.119 | 38.6 (7.3 to 73.8) | 43.3 (14.9 to 74.6) | 46.8 (22.3 to 73.6) |
|  | Water | 21.3 (15.3 to 27.2) | 23.0 (14.2 to 31.7) | 27.1 (19.9 to 34.2) | 0.220 | 408 (195 to 733) | 228 (108 to 386) | 162 (98 to 240) |
| Mean expenditure (2015/16 T$ /person/year) | Soft drinks | 3.7 (-13.0 to 20.4) | -1.7 (-20.7 to 17.3) | -26.4 (-51.0 to -1.8) | 0.047 | 18.3 (-62.6 to 106.0) | -5.0 (-58.5 to 55.3) | -39.0 (-60.8 to -9.6) |
|  | Milk | 6.7 (0.0 to 13.3) | 8.3 (-0.4 to 17.0) | 30.4 (8.1 to 52.7) | 0.046 | 61.4 (-3.8 to 139.4) | 42.1 (-4.3 to 95.7) | 80.9 (16.8 to 154.4) |
|  | Water | 3.9 (-5.2 to 13.1) | 17.6 (5.0 to 30.1) | 40.0 (25.3 to 54.7) | 0.000 | 73 (225 to -1,670) | 528 (109 to 1,106) | 353 (153 to 648) |
| Beverage expenditure as a proportion of household food budget (%) | Soft drinks | -0.02 (-0.46 to 0.41) | 0.21 (-0.61 to 1.02) | -0.30 (-0.72 to 0.12) | 0.361 | -2.0 (-37.2 to 37.8) | 14.4 (-40.6 to 78.5) | -16.8 (-37.5 to 5.9) |
|  | Milk | 0.52 (0.19 to 0.86) | 0.81 (0.27 to 1.35) | 1.38 (0.78 to 1.98) | 0.014 | 85.4 (22.2 to 162.3) | 91.6 (24.2 to 170.1) | 129.2 (65.6 to 200.6) |
|  | Water | 0.62 (0.35 to 0.88) | 1.66 (-0.25 to 3.56) | 1.20 (0.74 to 1.65) | 0.030 | 884 (340 to 1,748) | 1,088 (-154 to 2,662) | 280 (96 to 581) |
| Beverage expenditure per person as a proportion of equivalised income (%) | Soft drinks | -0.02 (-0.40 to 0.35) | -0.04 (-0.23 to 0.16) | -0.09 (-0.18 to 0.00) | 0.750 | -4.1 (-61.4 to 63.5) | -10.4 (-57.8 to 45.2) | -29.1 (-50.5 to -2.9) |
|  | Milk | 0.14 (-0.05 to 0.33) | 0.10 (0.00 to 0.20) | 0.16 (0.07 to 0.25) | 0.859 | 44.3 (-18.4 to 130.4) | 47.0 (-0.8 to 103.2) | 98.4 (35.9 to 169.8) |
|  | Water | 0.08 (-0.19 to 0.34) | 0.19 (0.05 to 0.34) | 0.20 (0.14 to 0.27) | 0.360 | 48 (134 to -1,171) | 495 (86 to 1,066) | 439 (220 to 744) |

All values are survey weighted to be representative of the national Tongan population. Absolute differences were calculated from the difference between the two means, with t-tests used to give the 95% confidence interval (CI) for the absolute difference and the p-value. Percentage change was calculated using the Kohavi method ^(2)^. The absolute difference p-value tests the null hypothesis that the absolute changes in outcome were the same in each sub-group, comparing the highest and lowest income group. The difference (Diff.) p-value tests for whether there was any evidence against the null hypothesis that there was no change over time. The 2009 expenditure findings were increased to adjust for inflation between 2009 and 2015/16 (12.4% increase, using CPI data from the Department of Statistics). Household expenditure was then divided by the number of people in the household. Source: Tonga Department of Statistics.

### Expenditure changes within the 2015/16 survey

Soft drink expenditure outcomes all decreased in the quarter following the July 2016 SB tax increase compared to the nine months prior; namely prevalence in household purchasing (42% to 34% of households, p=0.04), per capita household expenditure (33% to 20%, p=0.04), household food budget share (1.4% to 1.0%, p=0.03), household income share (0.6% to 0.4%, p=0.02) and quantity per person per year (9.2kg to 5.0kg, p=0.20) (Table S5).

There were trends of greater absolute declines in low-income households for purchasing prevalence and income share; and greater declines in high-income households for expenditure, food budget share and quantity (albeit not statistically significant). On the relative scale the declines were greatest in low-income households for all measures except for quantity which had the greatest decline for high-income households (Table S6).

Bottled water expenditure changes pre-post were small and not significant except for quantity which decreased by 39% (p=0.004). There was no evidence of differences in absolute changes in bottled water by income, except for quantity which decreased most in high-income households (p=0.01, Table S6).

We could not rule out the likely seasonal impacts on these results. It appears all beverage categories decreased to some extent suggesting a possible seasonal pattern. Demographic differences were likely to be minimal (4 out of 5 regions were sampled in all 16 rounds across the calendar year).

**Supplementary Table S5: Expenditure nine months before and three months after the 1 July 2016 tax change, which occurred three-quarters of the way through the conducting of the Tonga Household and Expenditure Survey, October 2015 to September 2016**

|  |  | **Pre-tax increase** | **Post- tax increase** | **Absolute difference** | **Expressed as a relative difference** | **p-value** |
| --- | --- | --- | --- | --- | --- | --- |
| Purchasing prevalence (% of households) | All beverages | 83.08 | 74.34 | -8.7 (-14.5 to -3.0) | -10.5 (-17.5 to -3.6) | 0.003 |
|  | Soft drinks | 42.47 | 34.41 | -8.1 (-15.7 to -0.4) | -19.0 (-37.1 to -0.9) | 0.040 |
|  | Milk | 46.09 | 39.90 | -6.2 (-14.6 to 2.3) | -13.4 (-31.7 to 4.9) | 0.151 |
|  | Water | 34.94 | 35.11 | 0.2 (-7.9 to 8.3) | 0.5 (-22.6 to 23.6) | 0.966 |
| Mean expenditure (2015/16 T$/person/year) | All beverages | 127.71 | 81.88 | -45.8 (-70.7 to -20.9) | -35.9 (-55.4 to -16.4) | <0.001 |
|  | Soft drinks | 32.81 | 20.39 | -12.4 (-24.2 to -0.6) | -37.9 (-73.8 to -1.9) | 0.039 |
|  | Milk | 39.19 | 24.40 | -14.8 (-25.1 to -4.5) | -37.7 (-64.1 to -11.4) | 0.005 |
|  | Water | 28.35 | 21.35 | -7.0 (-17.8 to 3.8) | -24.7 (-62.9 to 13.5) | 0.205 |
|  | All food and beverages | 2203.82 | 2001.43 | -202.4 (-475.9 to 71.1) | -9.2 (-21.6 to 3.2) | 0.147 |
| Beverages as a proportion of household food and beverage expenditure (%) | All beverages | 5.83 | 4.18 | -1.65 (-2.91 to -0.39) | -28.3 (-49.8 to -6.7) | 0.010 |
|  | Soft drinks | 1.44 | 0.97 | -0.5 (-0.9 to 0.0) | -32.8 (-63.1 to -2.5) | 0.034 |
|  | Milk | 1.80 | 1.35 | -0.4 (-0.9 to 0.0) | -24.8 (-50.0 to 0.4) | 0.054 |
|  | Water | 1.41 | 1.03 | -0.4 (-1.2 to 0.4) | -26.9 (-84.8 to 31.0) | 0.363 |
| Expenditure as a proportion of income (%) | All beverages | 2.27 | 1.59 | -0.7 (-1.2 to -0.1) | -30.1 (-54.0 to -6.1) | 0.014 |
|  | Soft drinks | 0.60 | 0.35 | -0.2 (-0.5 to 0.0) | -41.4 (-77.5 to -5.4) | 0.024 |
|  | Milk | 0.62 | 0.44 | -0.2 (-0.3 to 0.0) | -29.1 (-51.2 to -7.1) | 0.010 |
|  | Water | 0.41 | 0.37 | 0.0 (-0.3 to 0.2) | -11.0 (-60.9 to 38.9) | 0.664 |
| Estimated quantity of beverages purchased (kg/person/year) | All beverages | 11.34 | 6.75 | -4.6 (-8.2 to -0.9) | -40.4 (-72.7 to -8.2) | 0.014 |
|  | Soft drinks | 9.20 | 4.98 | -4.2 (-10.6 to 2.2) | -45.9 (-115.6 to 23.8) | 0.197 |
|  | Milk | 30.28 | 19.50 | -10.8 (-23.4 to 1.8) | -35.6 (-77.3 to 6.1) | 0.094 |
|  | Water | 62.21 | 38.17 | -24.0 (-40.3 to -7.7) | -38.6 (-64.8 to -12.5) | 0.004 |

The p-value here tests whether there was any difference in expenditure outcomes over time. We cannot rule out seasonal impacts on these results, demographic differences eg, in regions, or the impact of pre-existing trends and other time-varying confounding. It appears all beverage categories decrease to some extent suggesting a possible seasonal pattern or by region of roll out.

**Supplementary Table S6:** **Expenditure before and after the July 2016 tax change, which occurred three-quarters of the way through the conduct of the 2015/16 Tonga Household and Expenditure Survey**

|  |  | **Absolute difference by per capita household income tertile** | | |  | **Expressed as a relative difference**  **(% change)** | | |
| --- | --- | --- | --- | --- | --- | --- | --- | --- |
|  |  | Low | Middle | High | *p-value* | Low | Middle | High |
| Purchasing prevalence (% of households) | All beverages | -12.36 | -5.99 | -9.06 | 0.648 | -15.7 | -7.0 | -10.7 |
|  | Soft drinks | -8.63 | -9.95 | -6.65 | 0.819 | -23.8 | -20.5 | -15.8 |
|  | Milk | 2.78 | -8.67 | -10.56 | 0.152 | 7.8 | -18.1 | -20.1 |
|  | Water | -7.27 | 11.47 | -5.00 | 0.798 | -23.5 | 37.3 | -12.0 |
| Mean expenditure (2015/16 T$/person/year) | All beverages | -26.31 | -21.16 | -76.70 | 0.059 | -40.9 | -20.7 | -38.5 |
|  | Soft drinks | -10.37 | -7.83 | -16.52 | 0.642 | -62.6 | -27.1 | -33.9 |
|  | Milk | -3.68 | -8.07 | -26.71 | 0.067 | -24.6 | -27.0 | -40.4 |
|  | Water | -3.34 | 5.17 | -19.19 | 0.144 | -32.5 | 29.1 | -37.2 |
|  | All food and beverages | -121.72 | -37.36 | -230.57 | 0.701 | -8.9 | -2.1 | -7.2 |
| Beverages as a proportion of household food and beverage expenditure (%) | All beverages | -1.05 | -1.09 | -2.52 | 0.303 | -25.0 | -20.2 | -33.6 |
|  | Soft drinks | -0.44 | -0.38 | -0.57 | 0.803 | -42.6 | -26.1 | -32.7 |
|  | Milk | -0.03 | -0.40 | -0.74 | 0.207 | -3.1 | -23.8 | -30.5 |
|  | Water | -0.28 | 0.20 | -0.95 | 0.462 | -36.6 | 19.9 | -41.7 |
| Expenditure as a proportion of income (%) | All beverages | -1.22 | -0.44 | -0.58 | 0.342 | -37.6 | -20.4 | -35.6 |
|  | Soft drinks | -0.44 | -0.21 | -0.15 | 0.212 | -56.2 | -33.7 | -34.8 |
|  | Milk | -0.19 | -0.21 | -0.16 | 0.849 | -25.7 | -32.2 | -32.5 |
|  | Water | -0.19 | 0.16 | -0.16 | 0.860 | -40.7 | 44.9 | -38.9 |
| Estimated quantity of beverages purchased (kg/person/year) | All beverages | -3.90 | -2.56 | -6.45 | 0.527 | -64.4 | -26.4 | -38.3 |
|  | Soft drinks | -3.99 | -2.17 | -6.27 | 0.770 | -43.2 | -29.8 | -57.7 |
|  | Milk | -3.07 | 6.33 | -31.06 | 0.020 | -34.0 | 38.9 | -52.7 |
|  | Water | -12.03 | -0.95 | -51.76 | 0.014 | -42.1 | -2.3 | -48.9 |

The p-value here tests whether there were differences in the pre-post tax changes in expenditure between high and low-income households.

### Expenditure volume patterns in 2015/16

Table S7 describes the beverage quantities in 2015/16 estimated from expenditure by demographic factors. Acquired quantities of soft drink per person were significantly greater in adult only, high-income, bottled water (using main drinking water source), Tongatapu and university-educated adult households. Milk and water expenditure followed the same pattern, with the addition that greater quantities of water were acquired in households where all adults were obese. The picture for juice/other was more mixed, but there were significantly greater per capita quantities acquired in outer island households and no qualification households. In Tables 3B and 3C, similar patterns were evident with greater expenditure and food budget shares for soft drinks, milk and water in the adult only, high-income and Tongatapu households in 2015/16. For example, there was approximately fourfold greater soft drink expenditure in high- compared to low-income households. The picture for juice was again more mixed with greater expenditure and budget proportions in outer island households.

**Supplementary Table S7: Estimated quantities of annual beverages acquired (kg) per household member, 2015/16 Tonga household income and expenditure survey**

|  |  | **Soft drinks** | **Milk** | **Water** | **All beverages** |
| --- | --- | --- | --- | --- | --- |
| All households | Sum purchases | **10.2 (7.9 to 12.4)** | **10.3 (8.3 to 12.2)** | **27.5 (20.7 to 34.4)** | **56.1 (46.6 to 65.5)** |
|  |  |  |  |  |  |
| Age composition | Adults only | 15.9 (9.4 to 22.3) | 16.2 (11.3 to 21.2) | 43.4 (27.7 to 59.2) | **80.6 (61.3 to 99.8)** |
|  | Adults and children | 7.9 (6.5 to 9.4) | 7.9 (6.5 to 9.3) | 21.3 (15.4 to 27.2) | **46.5 (37.5 to 55.5)** |
|  | *P* | *0.017* | *0.001* | *0.005* | *0.001* |
|  |  |  |  |  |  |
| Income per person | Lowest income | 4.4 (2.8 to 6.0) | 3.9 (2.9 to 5.0) | 9.8 (5.4 to 14.2) | **26.4 (17.9 to 34.8)** |
|  | Middle-income | 9.3 (6.0 to 12.7) | 8.1 (6.2 to 10.1) | 16.3 (11.0 to 21.6) | **40.5 (31.2 to 49.7)** |
|  | Highest income | 15.4 (11.0 to 19.9) | 17.1 (12.6 to 21.7) | 51.7 (37.2 to 66.2) | **93.7 (75.0 to 112.3)** |
|  | *p (test for trend)* | *<0.001* | *<0.001* | *<0.001* | *<0.001* |
|  |  |  |  |  |  |
| Obesity (adults in the household) | All adults obese | 11.8 (7.5 to 16.0) | 12.3 (9.4 to 15.1) | 44.4 (28.9 to 60.0) | **73.8 (56.3 to 91.3)** |
|  | Mixed for obesity | 7.4 (5.8 to 9.0) | 8.0 (6.4 to 9.6) | 19.5 (14.5 to 24.4) | **45.3 (35.2 to 55.3)** |
|  | No adults obese | 19.5 (8.0 to 31.0) | 15.8 (4.1 to 27.5) | 20.2 (10.6 to 29.8) | **60.3 (40.6 to 80.0)** |
|  | *p (test for trend)* | *0.577* | *0.999* | *0.003* | *0.044* |
|  |  |  |  |  |  |
| Main drinking water source | Bottled water | 27.2 (12.1 to 42.4) | 15.7 (11.1 to 20.3) | 70.4 (45.9 to 95.0) | **118.3 (87.8 to 148.8)** |
|  | Tank or other supply | 8.7 (7.0 to 10.3) | 9.8 (7.7 to 11.8) | 23.8 (17.0 to 30.6) | **50.7 (41.1 to 60.2)** |
|  | *p* | *0.016* | *0.019* | *<0.001* | *<0.001* |
|  |  |  |  |  |  |
| Island | Tongatapu | 12.0 (8.9 to 15.0) | 12.5 (9.8 to 15.1) | 35.7 (26.3 to 45.1) | **62.3 (50.9 to 73.7)** |
|  | Outer island | 5.6 (3.9 to 7.3) | 4.6 (3.4 to 5.8) | 6.8 (4.3 to 9.3) | **40.2 (23.8 to 56.7)** |
|  | *p* | *<0.001* | *<0.001* | *<0.001* | *0.032* |
|  |  |  |  |  |  |
| Quarter | Oct-Dec 2015 | 13.5 (7.9 to 19.2) | 12.7 (8.0 to 17.5) | 19.4 (11.2 to 27.6) | **52.5 (38.4 to 66.6)** |
|  | Jan-Mar 2016 | 10.8 (6.9 to 14.8) | 14.0 (10.2 to 17.7) | 57.3 (30.8 to 83.8) | **101.3 (67.1 to 135.4)** |
|  | Apr-Jun 2016 | 8.6 (6.1 to 11.2) | 7.6 (5.5 to 9.7) | 25.8 (14.9 to 36.8) | **47.3 (34.5 to 60.0)** |
|  | Jul-Sep 2016 | 6.8 (4.5 to 9.0) | 6.9 (5.3 to 8.6) | 19.5 (10.4 to 28.6) | **38.2 (27.1 to 49.2)** |
|  |  |  |  |  |  |
| Highest qualification for head of household | Bachelor or post-grad | 26.3 (2.7 to 49.9) | 24.9 (14.9 to 34.9) | 68.0 (24.3 to 111.8) | **123.6 (70.0 to 177.1)** |
|  | Certificate or diploma | 10.5 (7.0 to 14.0) | 16.2 (8.1 to 24.4) | 49.7 (25.5 to 73.8) | **80.4 (52.6 to 108.1)** |
|  | Secondary sch. cert. | 10.7 (8.1 to 13.3) | 11.1 (8.5 to 13.7) | 28.5 (18.0 to 39.0) | **54.6 (42.2 to 67.0)** |
|  | No qualification | 8.5 (5.3 to 11.8) | 6.9 (5.3 to 8.5) | 17.6 (12.2 to 23.0) | **45.0 (33.7 to 56.4)** |
|  | *p (test for trend)* | *0.103* | *<0.001* | *0.001* | *0.001* |

Note: n=1803 Column p-values were calculated from survey weighted linear regression with quantity per person as the outcome, and the variable of interest as the explanatory variable. This was a test for trend when there were more two levels of the explanatory variable (ie, for income, obesity and highest qualification).

### Origin of imported beverages

The largest volumes of soft drinks came from Fiji (note gap in reporting in 2012), Malaysia, United States and New Zealand (Supplementary Fig. S1). There was a large and steep increase in import volumes from Malaysia from 2007 to 2011, and 2013 to 2015. At the same time import volumes from the United States and New Zealand markedly declined. The import price in US$ per litre varied by country and was greater for imports from New Zealand (US$1.31) and the United States (US$0.81) but beverage were cheaper from Fiji (US$0.50) and Malaysia (US$0.54).

**Supplementary Figure S1: Exports of sweetened beverages (HS 2202) to Tonga as reported by country of origin, 1990 to 2016**

Data source: UN Commodity Trade Statistics Database, Department of Economic and Social Affairs/Statistics Division, http://comtrade.un.org, Accessed 15 August 2018

## Further discussion

Sweetened drink import volumes in an earlier Tonga study (Teng et al. unpublished results) were approximately 42L per person a year in 2015/16, much larger than the estimated 10kg per person measured in the 2015/16 expenditure survey. Expenditure volume estimates may have been lower because of the narrower focus on soft drinks, exclusion of purchases consumed outside the home, undercounting and estimated quantities using price data. The estimated annual per person quantities of milk (10kg) and juice/other (8kg) expenditure in 2015/16 were not too dissimilar to the import volumes of milk (12L) and juice (4L) during the same time period (Teng et al. unpublished results).

In the same time period that the expenditure survey recorded soft drink expenditure declines, Tonga import volumes of sweetened beverages per person increased from an average of approximately 3L each month in 2009 to 4L a month in 2015/16 (Teng et al. unpublished results). Differing trends between expenditure volumes (decreased based on Coca-Cola 600ml price, see Discussion) and import volumes may result from increasing imports of other sweetened beverage products (not soft drinks), declines in the average soft drink price over time and the inability of the expenditure survey to capture soft drink expenditures in Tonga that may be increasing eg, by tourists and by children at school.

The pattern of increasing milk expenditure is consistent with previous increasing milk import volumes for Tonga ^(3)^, however large increases in water purchasing is not reflected in import volumes, suggesting that the rapid expansion local water bottling is the key driver of this trend. There was a large increase in households relying on bottled water as their main drinking water source from 2% to 8% similar to census findings ^(4, 5)^.

### Further strengths and limitations of this study

The survey populations differed somewhat with fewer people being surveyed during the Christmas quarter in 2015/16 which may have slightly underestimated expenditure. Conversely, underreporting of expenditure may have improved over time. For example, 28% of households using bottled water as their main drinking water source reported water expenditure in 2009, but this was 51% in 2015/16; but also possibly due to increased frequency of bottled water expenditure. Also soft drinks only make up a proportion of SBs that were taxed and thus results here may not fully represent the effects on other taxed beverages.

Other external factors may have affected study results for example flow-on impacts of the global financial crisis of 2007-2008 on Tonga’s economy, changes in GDP per capita (and differences by income group), changes in international visitor numbers (although these were low) and fluctuations in exchange rates.

## References

1. United Nations Commodity Trade Statistics Database - Department of Economic and Social Affairs/Statistics Division (2018) UN Comtrade data. <http://comtrade.un.org> (accessed August 15 2018)

2. Kohavi R, Longbotham R, Sommerfield D *et al.* (2008) Controlled experiments on the web: survey and practical guide. *Data Min Knowl Disc* 18, 140-181.

3. Teng AM, Jones AC, Mizdrak A *et al.* (2019) Impact of sugar-sweetened beverage taxes on purchases and dietary intake: Systematic review and meta-analysis. *Obes Rev* 20, 1187-1204.

4. Tonga Statistics Department (2017) *Tonga 2016 census of population an housing. Volume 1: Basic tables and administrative report*. Nuku’alofa, Tonga.

5. Statistics Department Tonga (2013) *Tonga 2011 census of population and housing. Volume 1: Basic tables and administrative report*.
